# Aesthetic comparison of the ideal nasal radix height in brazilians

**DOI:** 10.1590/S1808-86942011000300011

**Published:** 2015-10-19

**Authors:** Geraldo Augusto Gomes, Shiro Tomita, Glaucio Serra Guimarães, Carla Freire de Castro Lima, Manuela Salvador Mosciaro, Tiago Binotti Simas

**Affiliations:** 1 Master's degree in otolaryngology, Rio de Janeiro Federal University. Coordinator of the Esthetic and Functional Nose Surgery of the clementino Fraga Filho University Hospital, Rio de Janeiro Federal University; 2 Full professor of otorhinolaryngology, Rio de Janeiro Federal University; 3 Adjunct professor of orthodontics, Fluminense Federal University; 4 Undergraduate student, medical school, Rio de Janeiro Federal University; 5 Undergraduate student, Souza Marques Medical School; 6 Undergraduate student, Souza Marques Medical School

**Keywords:** anthropometry, beauty, surgery plastic, esthetics, rhinoplasty

## Abstract

The harmony of the facial profile is widely influenced by the height and form of the nasal dorsum. A few millimeters can make the lateral view aesthetically more or less pleasing and adequate in a subject's face. Professionals working with facial aesthetics should focus not only on the surgical techniques for proposed outcomes, but also with the subtleties and subjectivity that characterize aesthetic concepts and judgment.

**Material and Methods:**

A prospective survey to evaluate the preferences of a group of healthcare professionals working with facial aesthetics, a group of fine artists, and lay people about the best nasal radix height; the survey involved comparing 3 different nasal radix heights using computer-altered photographs of women with measurements close to the Caucasian standard.

**Results and conclusion:**

The lowest position of the nasal radix - close to the height of the pupil - was preferred (53%), followed by the middle position (superior crease of the eye). The highest position, resembling classic Greek statues, was considered the worst. The authors aos evaluated the effect of age, gender, and educational level on the choice of the best and worst female profiles.

## INTRODUCTION

Harmony and symmetry are universal elements of beauty. Artists and healthcare professionals have studied essentially abstract themes such as harmony and the difference between beauty and ugliness throughout history, to find the most agreeable facial proportions.^1^

Reis^2^ (2006) studied subjective evaluations of the face and found that the nose was the most frequent element described as responsible for a non-esthetic aspect in esthetically challenged male and female subjects. The height of the nasal radix is fundamental when assessing the profile of individuals.

Subtleties in the size and shape of the nose may positively or negatively affect our esthetic judgment; these points have been widely debated and studied.^3^

Maneuvers that change the shape of the nose may be required even if the main purpose of surgery is to correct function, and will affect the face.^4^, ^5^

Throughout history, cultural, economic, social, and anthropological factors have influenced individual and collective feelings about facial esthetics, which are dynamic and unfit for static and final conclusions. The height of the nose is important when assessing the profile of individuals.^6^ Most of the published papers on surgery related to this topic has originated from the United States and Western Europe, and is based on the classical model described by Renaissance artists.^7^, ^8^

Graphic computation may be applied to generate changes in picture of faces and help judge esthetic results; this topic still generates much debate among professionals working with facial esthetics.^9^

Few studies have been carried out in Brazil to evaluate the impressions that Brazilians have about the proportions of profiles as applied to nasal esthetics. Thus, sequential and consistent studies about Brazilian concepts of beauty and harmony are needed, since our cultural influences differ from those of the North-American and European cultures.

Therefore, the purpose of this study was to:

1) Compare the opinions of a group of plastic artists, a second group of healthcare professionals with experience in facial esthetics, and a third group of subjects with no professionals experience in facial esthetics, about three possible variations of nasal radix height in the profiles of young women with similar proportions to those of the Caucasian model.
2) Identify whether factors such as age, sex, and education affect the choice of best or worse profiles.

## METHODS

A prospective inquiry was carried out. The institutional review board evaluated and approved the study (no. 021/08), which met the requirements for clinical studies of human beings.

The first step consisted of gathering 66 standardized pictures of right profiles of volunteers aged from 18 to 30 years. These images were assessed for:

a) Eligibility criteria:
  Eligible pictures were profiles that were subjectively harmonic and similar to the classical Caucasian female profile by consensus of the authors, based on a purely qualitative analysis, without measurements. Seventeen of the 66 available profiles met these criteria.
b) Exclusion criteria:
  The 17 images selected initially (eligibility criteria) were measured based on linear and angular measures described for classical facial proportions using the Image J 1.38X software (National Institute of Health, USA). Images with measurements not within the standards described in the Dallas Rhinoplasty Symposium, with a ± 10% tolerance for each measure, were excluded. After this procedure, 11 of the 17 profiles were considered eligible, of which six met our aims, qualitatively and quantitatively.

A second step involved manipulating the six images selected in the eligibility and exclusion criteria in the Adobe Photoshop version 7.0.1 software (Adobe®) to adjust the height of the nasal radix along the cranial-caudal axis into three heights: (1) regular, at the height of the tarsal fold of the upper eye lid; (2) high, above the tarsal fold of the upper eye lid to the height of the upper border of the eyebrow; (3) low, below the tarsal fold to the lowest point of the pupil. These changes were done without altering the remaining previously measured proportions.

**Figure 1:**
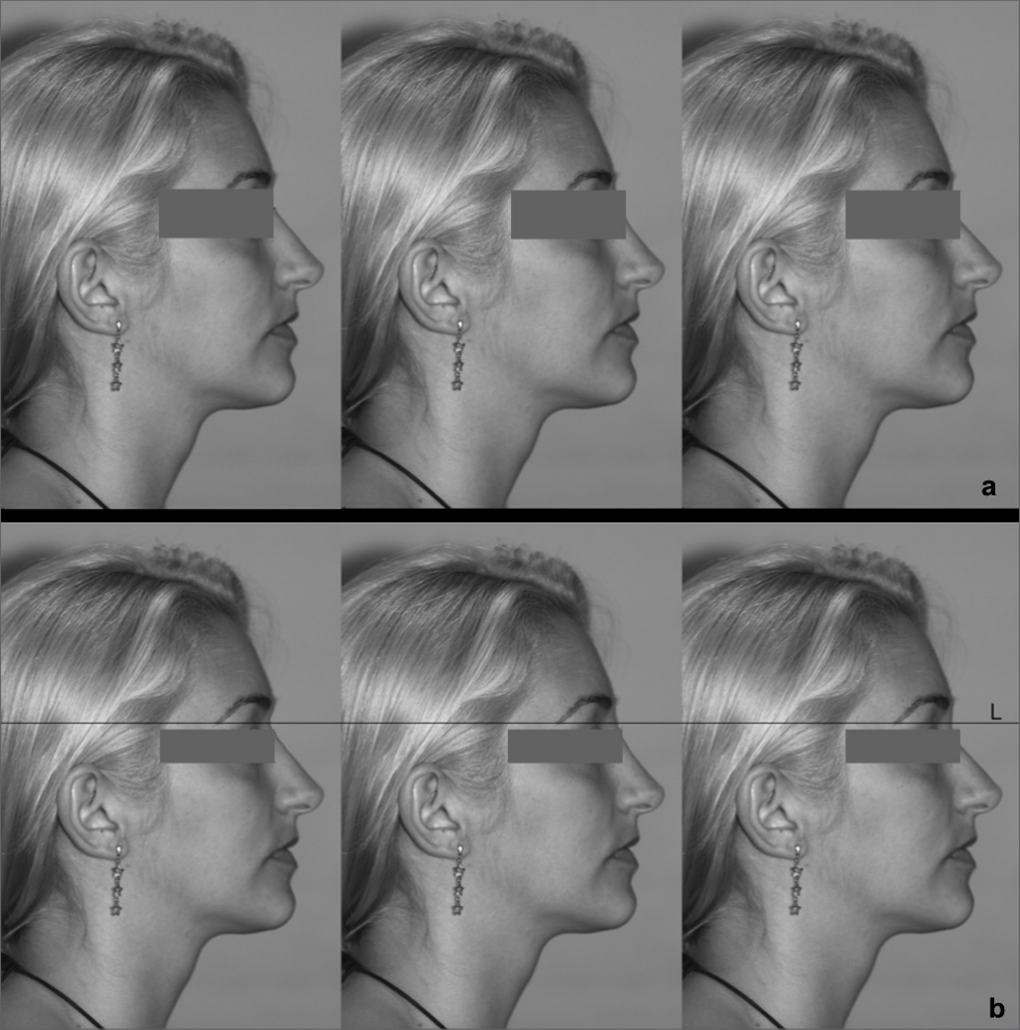
(a) Example of alignment of manipulated images, as shown to interviewees; from left to right: the regular profile, the higher profile, and the lower profile. (b) The same sequence with a reference line (L) representing the regular radix height (regular profile) to show readers the marked difference between the three heights of the nasal radix.

Thus, from each picture three electronically manipulated pictures were derived; each had either a profile with a high nasal radix (higher profile), a regular root (regular profile), and a lower root (lower profile), totaling six groups of three pictures each. Each picture was placed alongside other pictures randomly before being submitted to the evaluation of the interviewees, as shown in Figure 1.

The third step consisted of a critical evaluation by three persons with varying experience on this topic: (1) healthcare professionals with experience in esthetic surgery that involve changes in the profile of subjects; (2) university trained plastic artists - painting, drawing, and sculpture; (3) 60 male and 60 female subjects aged from 18 to 30 years with no experience in facial esthetics and at least of undergraduate level. Interviewees were asked which of three images based on the same profile were more pleasing (best) and which were least pleasing (worse). Age, sex, education, and occupation data of interviewees were recorded.

The EPIINFO/OMS2000 software was used to consolidate the data; the chi square test (χ^2^) was used for the analysis. The questionnaires were given during field work done by the main author and duly trained students of the Scientific Initiation Program.

## RESULTS

One hundred and sixty persons were interviewed. Each one assessed 6 image sequences, as shown in Figure 1, totaling 960 opinions. Each subject from three groups of individuals with varying interests in facial esthetics gave his or her opinion individually; these groups consisted of:

a) 120 opinions: twenty healthcare professionals working with surgery affecting the facial profile; seven otorhinolaryngologists, four bucco-maxillofacial surgeons, four plastic surgeons, and five orthodontists;
b) 120 opinions: twenty plastic artists (Fine Arts graduates) working regularly with drawing, painting, or sculpture of human faces; and
c) a group of lay persons comprising 60 male and 60 female subjects aged from 18 to 30 years, with no experience in human facial esthetics, and of at least undergraduate level.

There were 89 male (56%) and 71 female (44%) subjects in the full sample of 160 interviewees.

The mean age of interviewees was 29.4 years. The youngest were aged 18 years and the oldest was aged 80 years. The mode was 25 years.

The education level was 198 (28%) undergraduates and 522 (72%) graduates.

**Chart 1:**
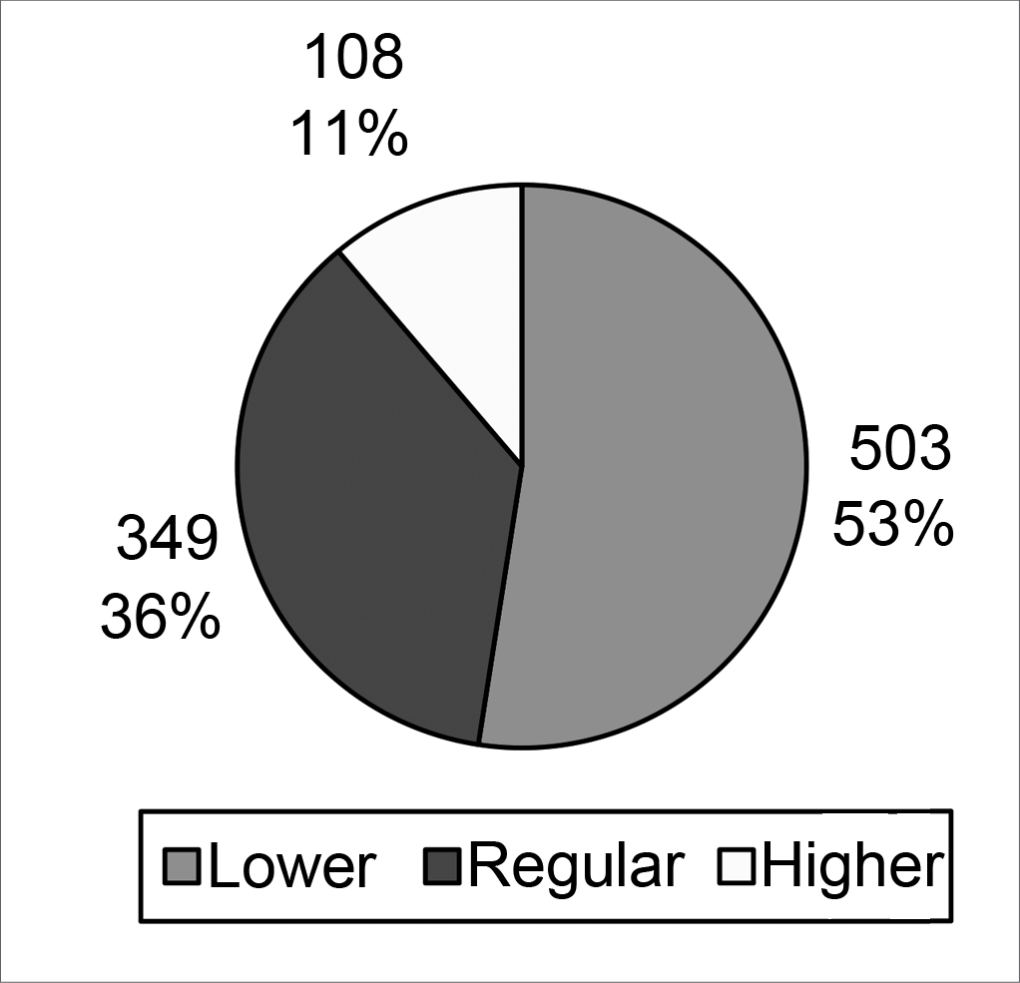
Best profiles without taking into account the group of professional experience to which interviewees belonged.

**Chart 2:**
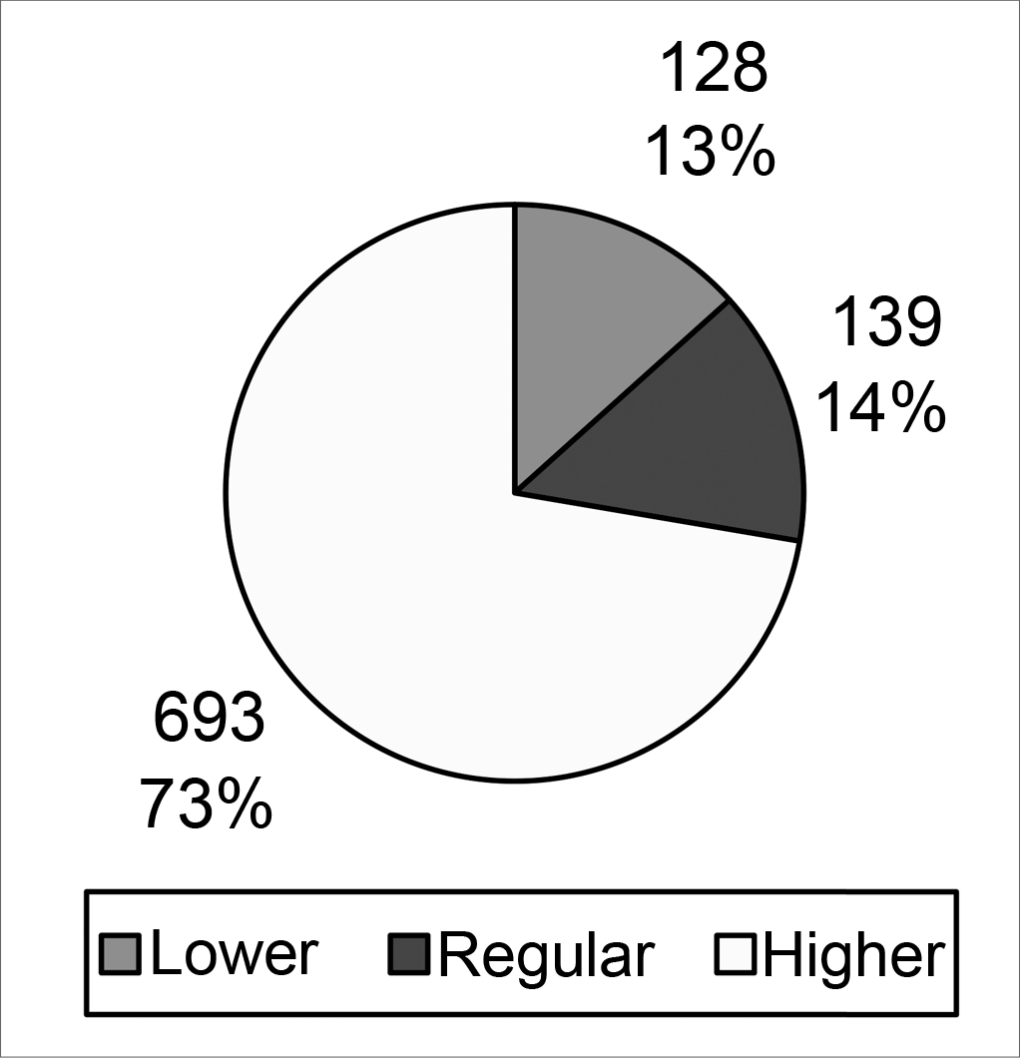
Worst profiles without taking into account the group of professional experience to which interviewees belonged

Charts 1 and 2 present the choice of profiles based on all opinions.

Tables 1 and 2 summarize the preferences according to profession.

**Table 1:** Best profile per interviewee group

	Lower	Regular	Higher	Total opinions
Professionals	60 (50%)	42 (35%)	18 (15%)	120 (100%)
Artists	82 (68%)	31 (26%)	7 (6%)	120 (100%)
Lay persons	361(50%)	276(38%)	83 (12%)	720 (100%)

*p* value = 0,003

**Table 2:** Worst profile per interviewee group

	Lower	Regular	Higher	Total opinions
Professionals	21 (18%)	18 (15%)	81 (67%)	120 (100%)
Artists	7 (6%)	10 (8%)	103(86%)	120 (100%)
Lay persons	100 (14%)	111 (15%)	509 (71%)	720 (100%)

*p* value = 0,007

Tables 3 and 4 present the results based on age groups.

**Table 3:** Best profile per age group of interviewees

Age group (years)	Best profile			
	Lower	Regular	Higher	Total
18-24	164(47,1%)	140(40,2%)	44(12,6%)	348(100%)
25-30	211(54,1%)	139(35,6%)	40(10,3%)	390(100%)
31-50	87(60,4%)	47(32,5%)	10(6,9%)	144(100%)
>50	41(52,6%)	23(29,5%)	14(17,9%)	78(100%)

**Table 4:** Worst profile per age group of interviewees

Age group (years)	Worst profile			
	Lower	Regular	Higher	Total
18-24	50(14,4%)	47(13,5%)	251(72,1%)	348(100%)
25-30	52(13,3%)	65(16,7%)	273(70%)	390(100%)
31-50	12(8,3%)	17(11,8%)	115(79,9%)	144(100%)
>50	14(17,9%)	10(12,8%)	54(69,2%)	78(100%)

**Chart 3:**
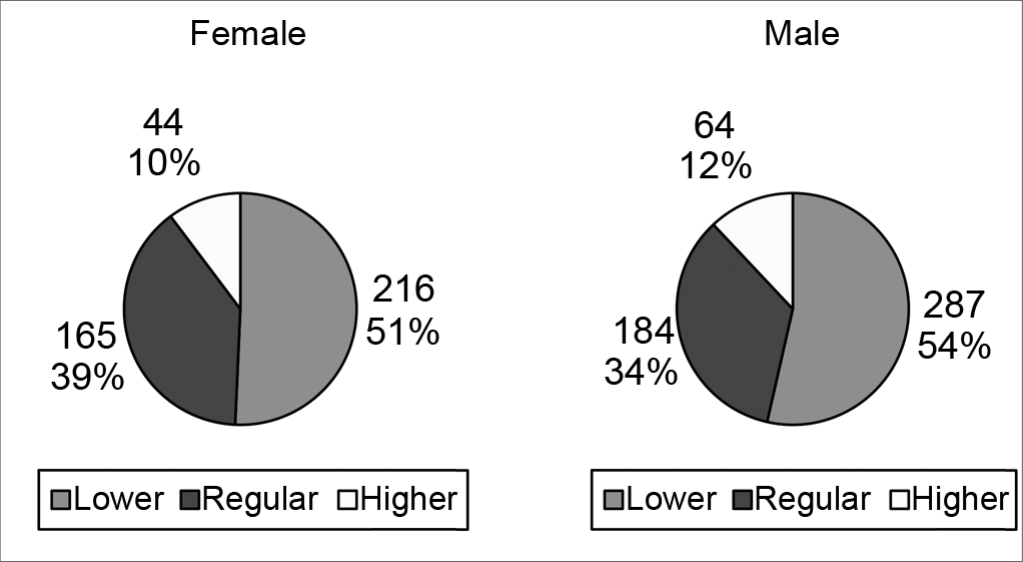
Best profiles according to the sex of interviewees.

**Chart 4:**
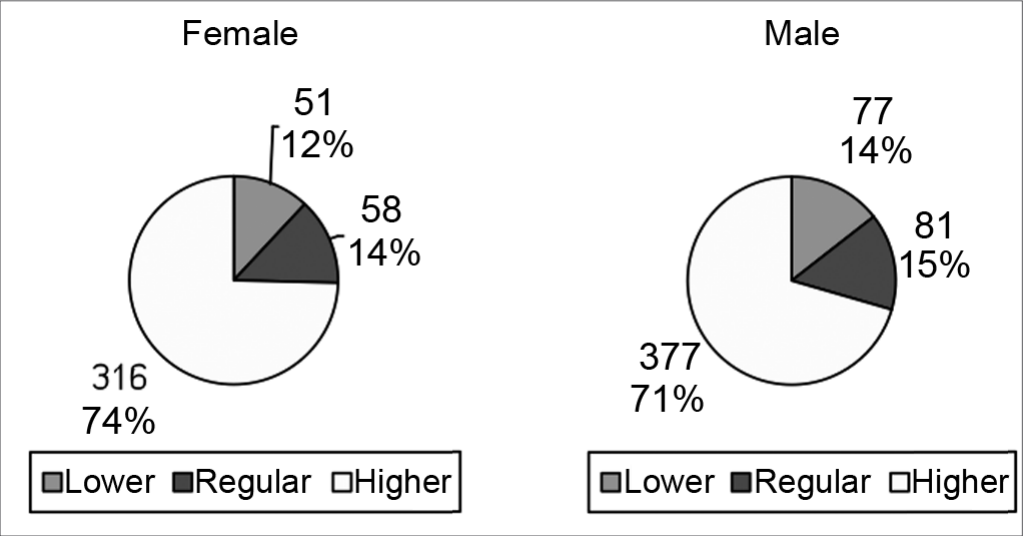
Worst profiles according to the sex of interviewees.

Charts 3 and 4 present the choices of best and worst profiles according to the interviewees' sex.

Tables 5 and 6 present the effect of education level on the choice of best and worst profiles.

**Table 5:** Best profile according to the education level of interviewees

Education level	Best profile		
	Lower	Regular	Higher
undergraduate	241 (46%)	219 (42%)	62 (12%)
graduate level	120 (60%)	57 (29%)	21(11%)

*p* value = 0,001

**Table 6:** Worst profile according to the education level of interviewees

Education level	Worst profile		
	Lower	Regular	Higher
undergraduate	81(16%)	77(15%)	364(69%)
graduate level	19(10%	34 (17%)	145 (73%)

*p* value = 0,109

## DISCUSSION

Beauty is in the eyes of the beholder. However, no study has characterized the average face of Brazilians, and few have attempted to establish local preferences in facial proportions and features.^2^ The paradox is that the demand for esthetic procedures and the development of this field of medicine in Brazil amply divulged internationally.

Plastic surgeons, otorhinolaryngologists, bucco-maxillofacial surgeons, and orthodontists daily seek to improve the esthetics of profiles. As mentioned above, subtleties of form may define the success or failure of esthetic treatments. Furthermore, it is key to understand the patient's expectations if the mutual satisfaction of patients and surgeons is to be met.

In spite of the efforts to provide a more objective perspective - evidenced in the citations of some authors in this paper - we find that such initiatives are considerable, but not sufficient, to approach the subject in depth.

In daily practice, subjectivity in facial esthetics brings a further challenge to professionals that seek the best treatment for their patients. Part of this results from the method of studies and statistics - used in most scientific papers - that look at sets of people, while medical practice deals with individuals, their tastes, and personal psychology. That is, even if results are one hundred percent aligned with scientific proposals, the results in some cases may not reach the expectations of patients. We decided to interview individuals that might enrichen our understanding about this topic from three perspectives: artists, from whom one expects a more sophisticated esthetic perception; lay persons, potential consumers of this knowledge; and healthcare professionals, who bring together technical information and esthetic judgment in their practice.

The analysis of opinions of the three groups about the question *“Which is the best profile?”* (Chart 1) revealed that the lower profile was preferred in 53% of cases, followed by the regular profile (36%), and the higher profile (11%). A 95% confidence interval was applied to clarify statistically whether the data suggested only a rejection of the higher profile or not; the numbers revealed that there was also a separation between the lower and regular profiles.

A statistical comparison based on the p value showed that opinion differences among groups (professionals, lay persons, and artists) were not random. The opinions of professionals and lay persons were similar - a preference for the lower profile in 50% of cases, followed by the regular profile, where both groups had similar percentages (professionals - 35%; lay persons - 38%). It appears to us that the opinions of professionals and lay persons is well attuned, which is beneficial when applying technical knowledge. The opinions of artists were more homogeneous - they preferred the lower profile in 68% of cases - and at a higher frequency compared to lay persons and professionals (Table 1).

Mowlavi^10^ (2004) published a similar study to ours in which he used standard drawings to compare opinions about male and female profiles focusing on the nasal radix; this author found significant rejection to the lower profile in a North-American study population. This contrasts with our findings, where a preference for the lower profile is suggested. This discrepancy is possibly due to cultural differences between US and Brazilian citizens.

Constantian^11^ studied 150 revision rhinoplasties (reoperations because of esthetic discontent) and showed that the main cause of disharmony - in 93% of cases - was a low nasal radix and/or low nasal dorsum. Confronting our results with those of Constantian^11^ and Mowlavi,^10^ we may think that within our culture lower profiles would be more acceptable. It seems, therefore, that a dorsum with a lower nasal radix is desirable for both surgeons and patients in the study group, in which the cultural influence is the same.

Although all interviewees were Brazilian, social, economic and cultural differences in different parts of the country may change the esthetic judgment. Thus, if we hypothetically compare our findings with those of a similar study done in another region of the country, there might be different opinions.

Even though most of the current literature recommends the regular height for the nasal radix, professionals in our study did not prefer this height; this concurs with Gunter's^12^ observation that esthetic judgment should be done case by case.

A comparison of the opinions of interviewees about the question *“what is the worst profile?”* showed that differences between opinions in each group were not statistically random (p value = 0.007). The higher profile was considered worst in most cases. Again, artists had a more homogeneous opinion; they rejected the higher profile 86% of times.

A study published in 2004^10^ showed that the higher profile was “tolerated” when males responded, but was rejected when female subjects were interviewed.

Webster et. al.^13^ suggests that a higher nasal radix with a shallow nasofrontal angle may have been desired by Romans and Greeks in ancient times, but it would be too masculine and unattractive for women. Peck and Peck^14^ underlined the fact that the tracings or men and women were treated identically in Greek art.

A higher nasal radix generates the impression that the nasofrontal angle is open and shallow, which amplifies the impression of a long nose and marked profile; this may be incompatible with a delicate image that some women prefer.

The influence of age on opinions shows that differences in the four age groups (18 to 24, 25 to 30, 31 to 50, and over 50 years) were always random. We found no studies that took age into account to compare our results. One of the few studies relating beauty and self-esteem - carried out in England - suggested that concerns with physical appearance in that population are more evident in the last adolescent years and the beginning of adult youth; this is when appearance is extremely important for relationships and social activities.^15^ The personality any individual desires affects his or her perceptions about what is attractive in the opposite sex.^16^ This same authors also stated that what people desire in the physical appearance of their partners reflects what they consider “good”; and that attractive faces are those that reflect these impressions.

The preferences of male and female subjects were similar with regards to the opinions about the best and worst profiles, both in the sample as a whole and when separated into each group of professionals.

Much attention from researchers in psychology and behavior has focused on debates about male and female preferences for certain facial features. Harris and Carr^15^ have pointed out a lack of knowledge about possible associations between sex, age, and the social and economic context on an understanding of the self-image. These authors have also stated that concerns with physical appearance were twice as high in women compared to men.

We restricted the education level our group of lay persons to undergraduates or above to improve the quality of opinions, based on Peck's^14^ statements in a paper published in 1970, where this authors found that the education level affected considerably a perception of the effect of esthetic deformities. So, we selected subjects with a higher education to decrease the possibility that lack of discernment could affect their critical judgment.

Although the effect of education was not our initial object of study, we found that there was a statistically significant difference between undergraduate and graduate subjects with regards to the question “*Which is the best profile?”*

Defining beauty has fascinated humankind for years. The topic is widely debated, but as we found in our study, several factors affect individual choices between what is pleasant and not pleasant, which precludes incontestable definitions of beauty.

Nevertheless, professionals are unquestionably responsible to their patients when committing themselves to increasing the harmony and beauty of their faces; these professionals then are obliged to seek an understanding of complex notions of beauty if they wish to offer their professional work towards this aim.

## CONCLUSION

The data suggest:

1) Significant rejection of the profile esthetics with the nasal radix positioned between the tarsal fold of the upper eyelid (higher profile) and the level of the upper border of the eyebrow.
2) Predominance of the profile below the tarsal fold to the lowest point of the pupil as the “best profile” in all analyses, contrasting with the current international literature.
3) Age and sex did not affect the subjects' choice.
4) Education of lay persons with higher education statistically altered the answer about the “best profile.”
